# Huppke–Brendel syndrome: Novel cases and a therapeutic trial with ketogenic diet and N‐acetylcysteine

**DOI:** 10.1002/jmd2.12439

**Published:** 2024-07-19

**Authors:** Katarina Šikić, Tessa M. A. Peters, Udo Engelke, Danijela Petković Ramadža, Tamara Žigman, Ksenija Fumić, Maša Davidović, Sanda Huljev Frković, Tibor Körmendy, Diego Martinelli, Antonio Novelli, Francesca Romana Lepri, Ron A. Wevers, Ivo Barić

**Affiliations:** ^1^ Department of Pediatrics University Hospital Center Zagreb Zagreb Croatia; ^2^ Donders Institute for Brain, Cognition and Behavior Radboud University Medical Center Nijmegen The Netherlands; ^3^ Department Human Genetics, Translational Metabolic Laboratory Radboud University Medical Center Nijmegen The Netherlands; ^4^ University of Zagreb, School of Medicine Zagreb Croatia; ^5^ Department of Laboratory Diagnostics University Hospital Centre Zagreb Zagreb Croatia; ^6^ Department of Diagnostic Neuroradiology University Hospital Centre Zagreb Zagreb Croatia; ^7^ Division of Metabolic Diseases, Department of Paediatric Subspecialties and Liver‐Kidney Transplantation Bambino Gesù Children's Hospital Rome Italy; ^8^ Translational Cytogenomics Research Unit Bambino Gesù Children's Hospital, IRCCS Rome Italy

**Keywords:** acetylated amino acids, acetyl‐CoA transporter, Huppke–Brendel syndrome, ketogenic diet, N‐acetylcysteine, protein acetylation

## Abstract

Huppke–Brendel syndrome (HBS) is an autosomal recessive disorder caused by *SLC33A1* mutations, a gene coding for the acetyl‐CoA transporter‐1 (AT‐1). So far it has been described in nine pediatric and one adult patient. Therapeutic trials with copper histidinate failed to achieve any clinical improvement. Here, we describe the clinical characteristics of two novel patients, one of them diagnosed by gene analysis and his sib postmortally based on clinical characteristics. We demonstrate a therapeutic trial with acetylation therapy, consisting of N‐acetylcysteine and ketogenic diet, in one of them. We provide biochemical data on N‐acetylated amino acids in cerebrospinal fluid (CSF) and plasma before and after starting this treatment regimen. Our results indicate that ketogenic diet and N‐acetylcysteine do not seem to normalize the concentrations of N‐acetylated amino acids in CSF or plasma. The overall metabolic pattern shows a trend toward lowered levels of N‐acetylated amino acids in CSF and to a lesser extent in plasma. Although there are some assumptions, the function of AT‐1 is still not clear and further studies are needed to better understand mechanisms underlying this complex disorder.

**Synopsis**

Here, we describe novel cases of Huppke–Brendel syndrome and demonstrate a novel therapeutic attempt with ketogenic diet and N‐acetylcysteine.

## 1 INTRODUCTION

Huppke–Brendel syndrome (HBS) (OMIM #614482) is an autosomal recessive disorder which was first described in 2005 by Horvath et al.^1^ Since then, it has been described in eight more pediatric^2^, ^3^, ^4^, ^5^ and one adult patient.^6^ It is caused by mutations in the *SLC33A1* gene (MIM #603690) coding for acetyl‐CoA transporter‐1 (AT‐1), a transmembrane protein located in the endoplasmic reticulum (ER) which regulates the influx of acetyl‐CoA from the cytosol into the lumen of the ER.^4^ AT‐1 is important for posttranslational protein modification together with acetyltransferases ATase1/NAT8B and ATase2/NAT8.^7^, ^8^ Studies outlining the function of AT‐1 are inconsistent.^3^ Main clinical characteristics of the syndrome include congenital bilateral cataracts, sensorineural hearing loss, and severe developmental delay, and the main laboratory findings are low serum copper and ceruloplasmin concentrations.^9^ Low concentrations of N‐acetylated amino acids in cerebrospinal fluid (CSF) and some in plasma were found in a previously published patient, diagnosed in our center. Using untargeted LC‐Qtof mass spectrometry, also referred to as next‐generation metabolic screening (NGMS), we described potential novel biomarkers for this complex disorder and a possible basis for intervention.^4^ So far, all therapeutic trials, including repeated attempts with copper histidinate failed to achieve any clinical improvement. All pediatric patients described in extenso, including the one we previously described, died before the age of 6 years. The only living adult patient, initially thought to have Wilson's disease, was treated for 25 years with zinc therapy.^6^ Here, we describe novel cases of HBS and demonstrate a novel therapeutic attempt with ketogenic diet and N‐acetylcysteine, which both provide acetyl‐CoA, based on our observation on an abnormal concentration of various N‐acetylated amino acids in CSF and plasma in our previously published patient.

## 2 CASE REPORT

Our patients were born from consanguineous parents of Roma ethnic origin. Two pregnancies ended with spontaneous miscarriages. Two older brothers and an older sister are healthy.

Our first patient was born from the 4th pregnancy at 39th week of gestation, with birth weight 2970 g (19th centile), birth length 49 cm (29th centile), head circumference 33.5 cm (24th centile), and Apgar scores 10 at 1 and 5 min. He had congenital bilateral cataracts, congenital hypotonia, perimembranous subaortic ventricular septal defect (VSD), red hair, dysmorphic facial features (anteverted nostrils, smooth philtrum, thin upper lip with downturned corners of the mouth, high‐arched palate without clefting), bilateral sensorineural deafness, and severe psychomotor retardation. At the age of 18 months brain magnetic resonance imaging (MRI) showed a posterior cranial fossa cyst and signs of dysmyelination and white matter reduction. During infancy, he had multiple severe respiratory infections. He died at the age of 3 years because of gastrointestinal bleeding of unknown etiology. After establishing genetic diagnosis in our second patient described below, we became aware that this patient also suffered from HBS.

**FIGURE 1:**
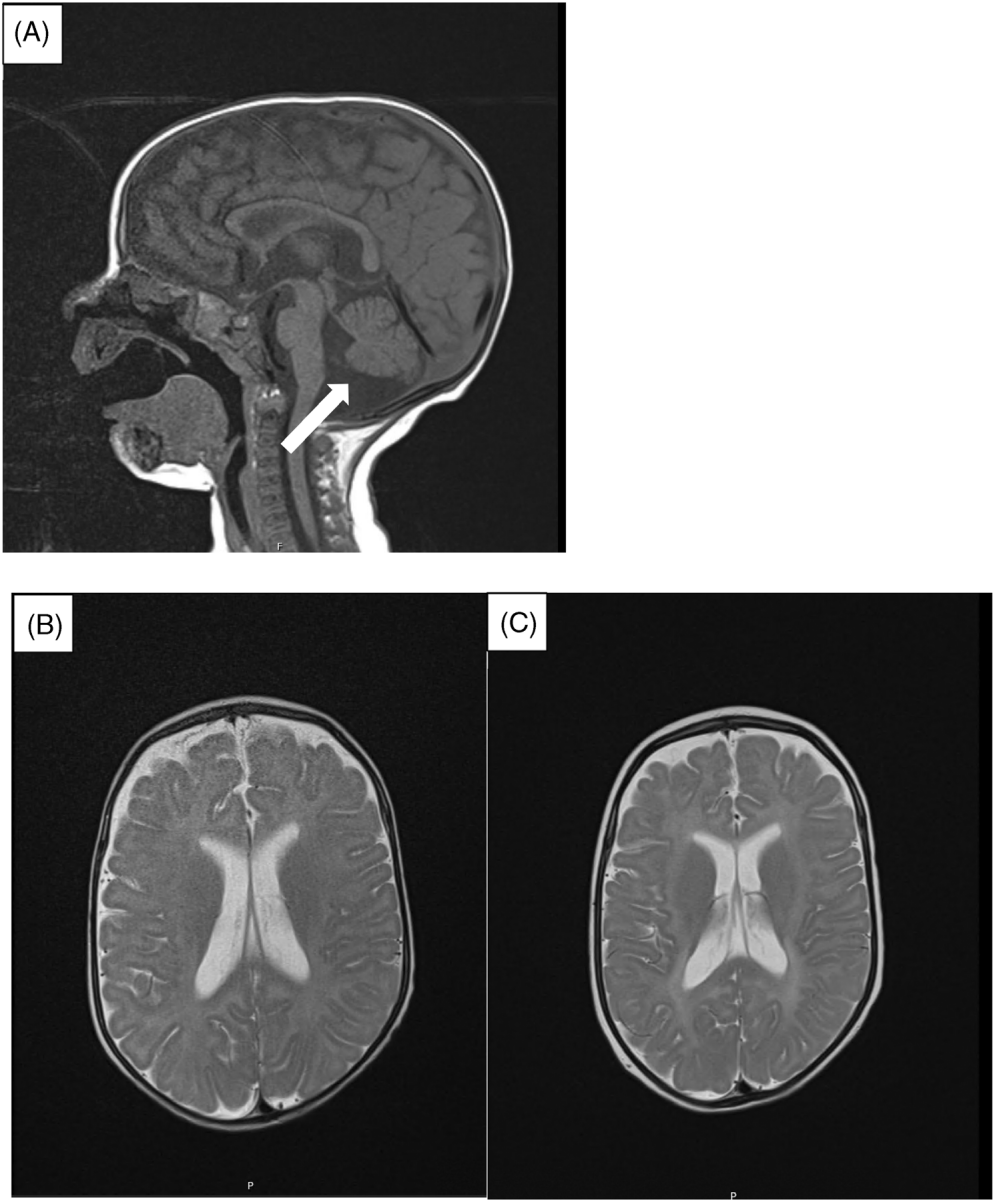
(A) T1W sagittal image showing hypoplasia of the caudal part of the cerebellar vermis (white arrow) at the age of 6 months. (B) and (C) T2W transversal image showing mild diffuse white matter hyperintensity, consistent with hypomyelination at the age of 6 months (B) and its progression at the age of 8.5 months (C).

Our second patient was born from mothers 7th pregnancy at 40th week of gestation, birth mass was 3090 g (15th centile), birth length 49 cm (17th centile), and head circumference 34 cm (23rd centile). Apgar score was 10 at 1 and 5 min. Upon birth he had congenital bilateral cataracts and dysmorphic features (micrognathia, cleft soft palate, smaller palpebral fissures, low‐set, small ears, low nasal bridge). He did not pass the newborn hearing test. He was hospitalized at our department for the first time at the age of 2 months, after the surgical treatment of incarcerated inguinal hernia. Brain MRI at that age was normal. Audiological work‐up revealed severe bilateral hearing impairment. He had perimembranous VSD and secondary hypothyroidism. He had low ceruloplasmin (0.03 g/L; ref. 0.16–0.45 g/L) and copper concentrations (2.0 μmol/L; ref. 11.3–28.0 μmol/L) while metabolic and other biochemical tests were normal. He was initially fed through a gastric tube with a satisfactory weight gain. The diagnosis of HBS was made by whole exome sequencing which revealed a homozygous variant c.775+ 1G>T in the *SLC33A1* gene. Based on the unresponsiveness of HBS patients so far to the therapy with copper histidinate and in view of the abnormal acetylation as a presumed pathogenesis of this disease, a therapy with N‐acetylcysteine 60 mg/kg/day in three daily doses and a ketogenic diet 3:1, introduced gradually over 5 days, was initiated. Unfortunately, after 33 days of therapy there was no clinical nor biochemical improvement. New brain MRI was performed at the age of 6 months, before this therapy regimen, and showed only slightly wider cisterna magna due to hypoplasia of the caudal part of the cerebellar vermis. The follow‐up imaging performed two and half months later, showed deterioration of the intracranial status, without further progress of myelination and with appearance of pathologic T2 hyperintensity of deep white matter and restriction of diffusion of axonal tracts of cerebrum and hippocampal/parahippocampal regions (Figure 1). There were no significant changes in brain MR spectroscopy before and after initiation of therapy. Considering all of this, we decided to discontinue the therapy. Our patient is now one and half year old, severely psychomotorically disabled, hypotonic, with severely impaired vision despite cataract surgery. His skin and his hair are hypopigmented. He is fed through a percutaneous gastrostomy tube. He had multiple severe infections, some with respiratory failure for which mechanical ventilation was needed. Partial closure of the perimembranous VSD is being monitored. His copper and ceruloplasmin concentrations are constantly low.

## 3 METHODS

In order to evaluate the effect of ketogenic diet and N‐acetylcysteine supplementation (acetylation therapy) samples of patient's plasma were taken before, 11 days after and 33 days after initiation of therapy. Initiation date is defined as the time point when ketogenic diet has reached the 3:1 ratio, that is, 13 days after start of N‐acetylcysteine. CSF samples were taken before and 33 days after initiation of acetylation therapy. We also measured copper and ceruloplasmin concentrations in patient's plasma before, 11 days after and 33 days after initiation of acetylation therapy.

### 3.1 Targeted analysis of N‐acetylated amino acids

Samples were analyzed in duplicate using UHPLC‐QTOF‐MS as previously described.^10^, ^11^ The raw data were analyzed in a targeted fashion to assess the N‐acetylated amino acids which were reported in our recent publication to be decreased (see Table S2 of Šikić et al^4^). The raw data belong to Patient #2 of this paper. The average intensities of duplicate samples were normalized to the mean QC sample intensity of the respective run.^4^ This allows comparison with previous results. Samples from the different time points were compared to assess the effect of acetylation therapy. Furthermore, samples from the patient reported in this manuscript (Patient 2) were compared to the samples of controls and of the patient described in our previous publication on HBS (Patient 1).^4^

## 4 RESULTS

### 4.1 Results of CSF analysis

**FIGURE 2:**
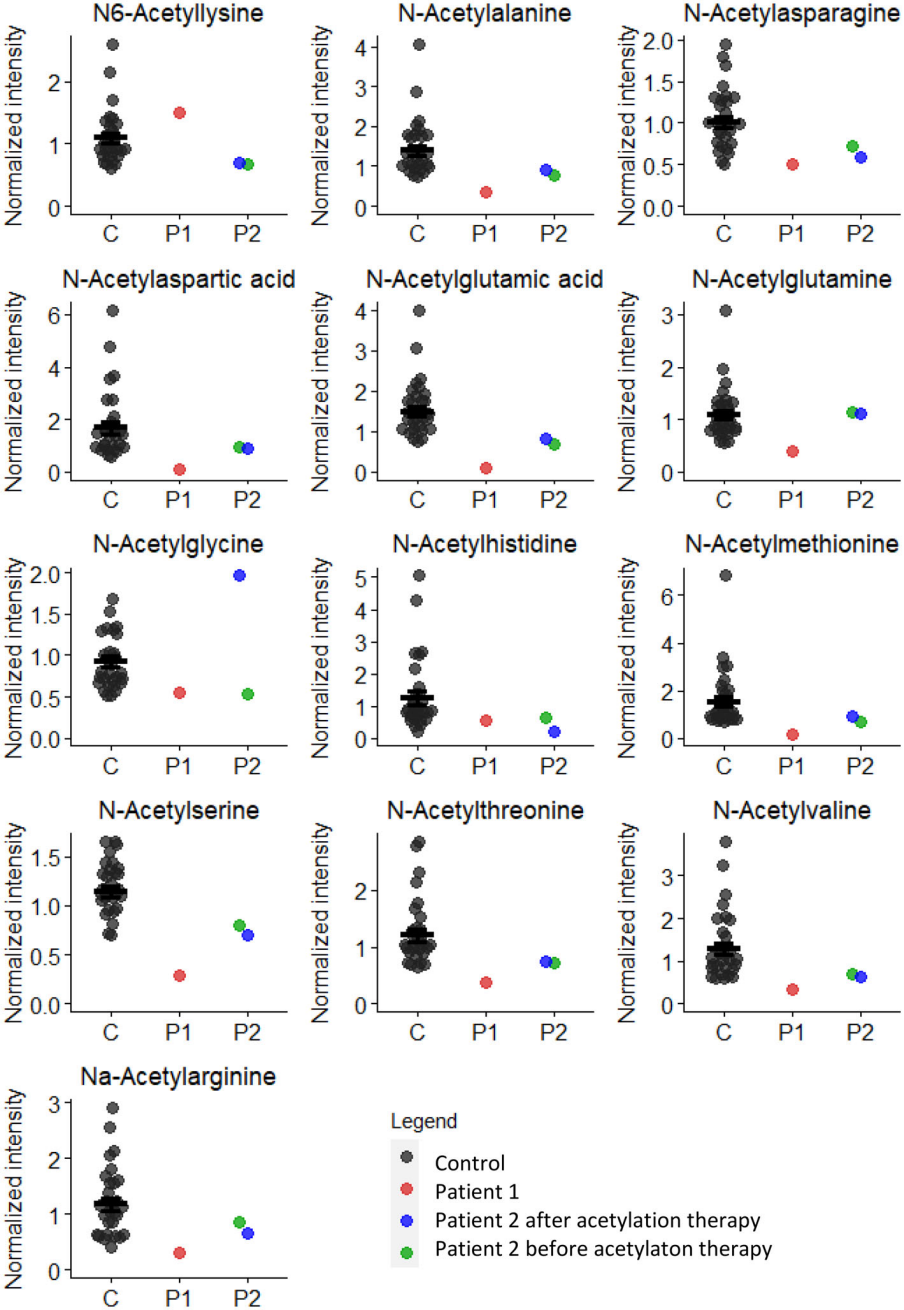
Normalized intensities of N‐acetylated amino acids in CSF samples from controls, Patient 1 (P1) and Patient 2 (P2). For P2, two samples are included: One before the start of acetylation therapy, and one 33 days after.

The results from the CSF samples of Patient 2 are shown in Figure 2. The intensities of almost all CSF N‐acetylated amino acids were in the low normal range in Patient 2. Of note, signals for Nα‐acetyllysine, N‐acetylphenylalanine, and N‐acetyltyrosine, which were described in Patient 1, were not quantifiable in Patient 2 and are therefore not included in the figure.

#### 4.1.1 N‐acetylated amino acids in CSF before and after acetylation therapy

Acetylation therapy did not significantly influence the concentrations of most N‐acetylated amino acids in CSF of Patient 2, except for that of N‐acetylglycine which is a known ketogenic diet biomarker (Figure 2).^12^ Its concentration increased from low normal before start of therapy to higher than the highest control in the sample on treatment. Other ketogenic diet biomarkers, 3‐hydroxybutyate and 3‐hydroxybutyrylcarnitine, also clearly increased during the ketogenic diet (data not shown).

#### 4.1.2 Results in Patient 2 in comparison to controls and Patient 1

The normalized intensities of N‐acetylated amino acids in CSF of Patient 2 were all within the range of the intensities of the control CSF samples, unlike the intensities in the CSF of Patient 1 (Figure 2). However, for most of the metabolites which were below the control range in Patient 1, intensities were on the lower end of the control range in Patient 2. This is the case for N‐acetylalanine, N‐acetylaspartic acid, N‐acetylglutamic acid, N‐acetylmethionine, N‐acetylserine, N‐acetylthreonine, N‐acetylvaline, and Nα‐acetylarginine. N‐acetylglutamine was below controls in Patient 1, but in Patient 2 showed an average intensity. For N‐acetylasparagine, N‐acetylglycine, and N‐acetylhistidine, both Patients 1 and 2 showed intensities in the lower end of the control range. Patient 2 also showed this for N6‐acetyllysine, while Patient 1 showed an above‐average intensity.

### 4.2 Results of plasma analysis

#### 4.2.1 N‐acetylated amino acids in plasma before and after acetylation therapy

**FIGURE 3:**
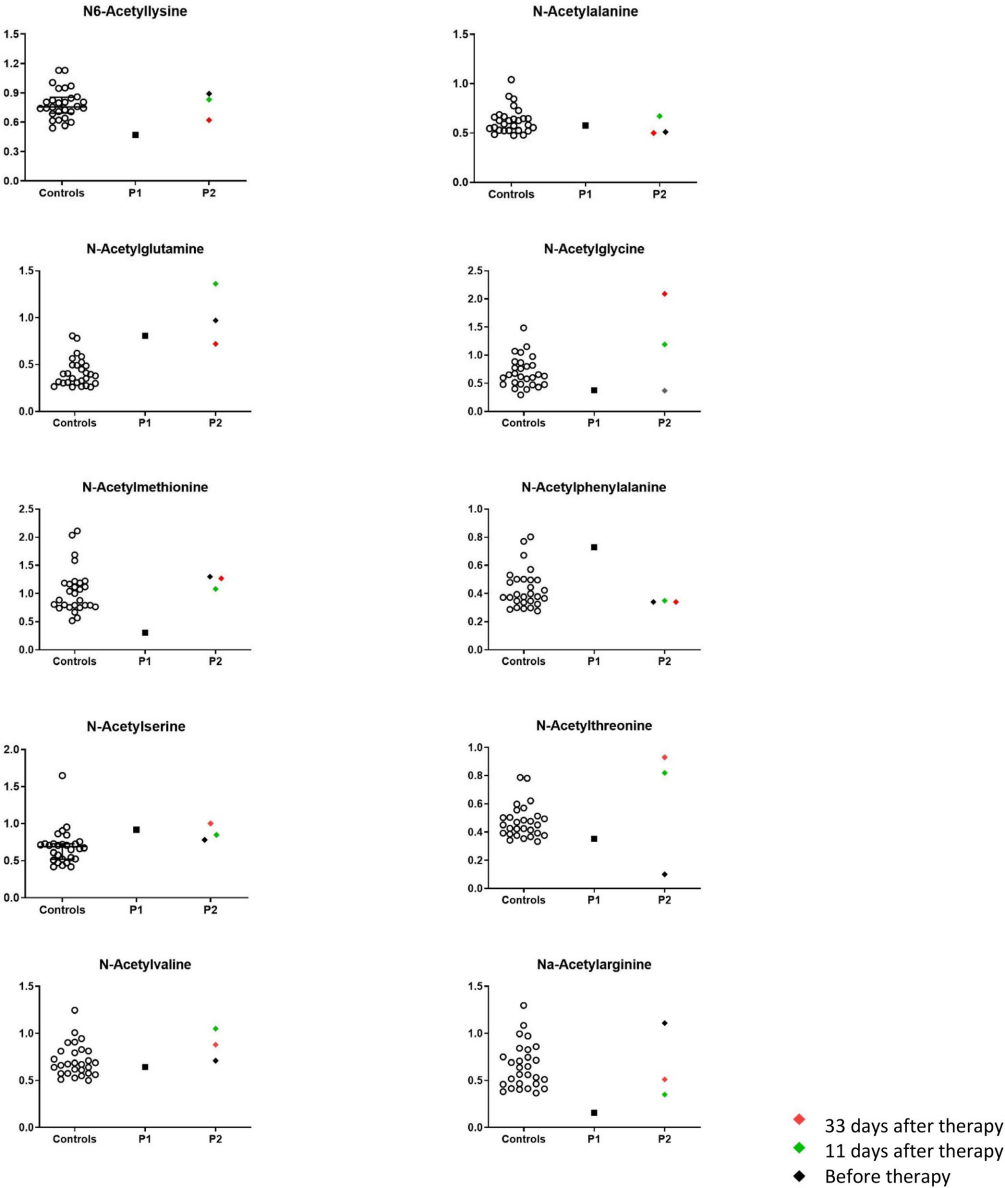
Normalized intensities of N‐acetylated amino acids in plasma samples from controls, Patient 1 (P1) and Patient 2 (P2). For P2, three samples are included: One before the start of acetylation therapy, one 11 days after, and one 33 days after.

The results for N‐acetylated amino acids in plasma samples are shown in Figure 3. For most metabolites, the results do not seem to be dependent on the timing with regard to the start of therapy. This is the case for N6‐acetyllysine, N‐acetylalanine, N‐acetylglutamine, N‐acetylmethionine, N‐acetylphenylalanine, N‐acetylserine, and N‐acetylvaline. For plasma and CSF N‐acetylglycine, a known ketosis biomarker, a gradual increase in intensity was observed in line with good compliance to the ketogenic diet. A similar increase is observed for the N‐acetylthreonine intensity in plasma while the intensity of this metabolite in CSF was not influenced by the therapy. We cannot conclude whether the change in plasma concentration was also due to the ketosis or depended on N‐acetylcysteine supplementation. Nα‐acetylarginine showed lower intensities after 11 and 33 days, than before therapy. Overall, there does not seem to be a normalizing effect on the plasma levels of N‐acetylated amino acids as a consequence of the acetylation therapy.

#### 4.2.2 Results in Patient 2 in comparison to controls and Patient 1

For most evaluated N‐acetylated amino acids in plasma, the intensities of Patient 2 were within the control range for all three time points. Exceptions occurred for N‐acetylglutamine, N‐acetylglycine, N‐acetylthreonine, and Nα‐acetylarginine. The intensities for N‐acetylglutamine are generally high in all samples of Patient 2 as well as in the sample of Patient 1. The high levels of N‐acetylglycine in the samples after acetylation therapy fit with literature describing this metabolite as a biomarker for ketogenic diet.^12^ N‐acetylthreonine shows an intensity below the control range before, and above this range 11 and 33 days after the start of acetylation therapy. For Nα‐acetylarginine, the 11th day sample is just below controls, but the 33rd day sample is within the normal range again.

The low levels of plasma Nα‐acetylarginine, N‐acetylmethionine, and N6‐acetyllysine which were previously described in Patient 1 are not present in Patient 2, while the low level of N‐acetylthreonine that is seen in Patient 2 was not present in Patient 1. In contrast to the results from CSF, there does not seem to be an overall trend of low‐normal intensities for Patient 2.

### 4.3 Ceruloplasmin and copper concentrations

Copper concentrations before, 11 days after and 33 days after acetylation therapy were 5.6, 3.8, 7.8 μmol/L, respectively. Ceruloplasmin concentrations before, 11 days after and 33 days after acetylation therapy were 0.06, 0.10, and 0.06 g/L, respectively.

## 5 DISCUSSION

HBS has been described in nine pediatric and one adult patient so far. Except for secondary hypothyroidism, which has not yet been described in patients suffering from HBS, our patient has all main clinical and laboratory features of this disorder. As copper histidinate therapy was of no or at best of questionable therapeutic benefit in so far treated patients, we considered a novel therapy by trying to provide more substrate for AT‐1 by giving acetylated compounds. Acetylsalicylic acid therapy was previously shown to affect protein acetylation.^13^ However, it would require long term use with potential side effects of this drug which was the reason why it was not our therapeutic choice.

Ketogenic diet is a well‐known therapy for some neurological disorders, primarily epilepsy, but also metabolic disorders.^14^ This high‐fat, low carbohydrate diet leads to overproduction of acetyl‐CoA derived from beta‐oxidation of fatty acids.^15^, ^16^ Experiments on mouse models with liver‐specific knock‐out pyruvate carboxylase (PcxL‐/‐) and consequently enhanced ketogenesis, showed mitochondrial protein hyperacetylation as the dominant signature, with minimal changes in acetylpeptide levels outside mitochondria.^17^ Considering this, we hypothesized that a ketogenic diet could be of benefit for the patient as it provides acetyl‐group. Another acetyl‐group providing drug, N‐acetylcysteine, was proven safe, orally widely bioavailable and efficient in children in multiple randomized clinical trials for conditions such as acetaminophen and non‐acetaminophen acute liver failure, autism spectrum disorders, psychiatric disorders etc.^18^, ^19^, ^20^ Therefore, to increase the chances of therapeutic success, we decided to perform a treatment trial with both ketogenic diet and N‐acetylcysteine.

Regarding our results, the metabolic fingerprint previously described in Patient 1 was not as clearly observed in Patient 2, although the overall pattern did show somewhat lowered levels of N‐acetylated amino acids in CSF. In the plasma of Patient 2, some deviations were observed, but these were not similar to those seen in Patient 1. There are probably a few explanations for this difference in intensities of some N‐acetylated amino acids between Patients 1 and 2. First, Patient 2 might have milder phenotype and therefore milder biochemical abnormalities. Patient 2 is homozygous for a splice site variant c.775+1G>T classified as pathogenic, while Patient 1 has a nonsense variant. Based on the clinical presentation, however, we cannot say that Patient 2 has a milder phenotype than Patient 1 who came to us at later age and was relatively better in infancy than at time of referral to us. Patient 2 has all features attributable to classical HBS and it is hard to say if he could be clinically assessed as milder. Another possibility is that abnormalities in some N‐acetylated amino acids were clearly recognizable in Patient 1 and not in Patient 2 because the sampling in Patient 1 was in more advanced disease phase. Patient 1 was 14 months old at the time of CSF sampling and Patient 2 was 6 months/8 months old. Another explanation could be a known decrease of concentration of many N‐acetylated compounds with age, with the especially strong effect in the first months/years of life. The controls we used are aged 0–2 years so both patients fall into this range. Unfortunately, we do not have subgroups within our 0–2 years old control cohort, and it would be valuable to study the 0 to 2 group in more detail.^11^

The ketogenic diet and the N‐acetylcysteine supplementation seemingly did not influence the intensities of the N‐acetylated amino acids significantly. As expected only CSF‐ and plasma‐N‐acetylglycine clearly increased on ketogenic diet, because this metabolite is known to be a biomarker for ketosis in a ketogenic diet setting.^12^ The clinical course during the acetylation therapy did not show any clearly positive changes, particularly considering the fact that this was during time when infants normally acquire some skills. As already mentioned, the follow‐up brain MRI after acetylation therapy showed deterioration of the intracranial status. Considering no positive effect on biochemical profile nor on clinical status, we decided to discontinue the therapy.

There has been some disagreement regarding the localization and function of AT‐1 and acetyltransferases. Huppke and his coworkers did experiments which showed a localization of AT‐1 in the cis‐, medial‐, and trans‐Golgi, and not in the ER as described previously by Puglielli and his team. Huppke explained the conflicting results were most probably due to the different methods used to determine the localization of AT‐1.^2^, ^21^ However, in later papers, Puglielli and coworkers confirmed the function of AT‐1 as ER membrane acetyl‐CoA transporter which works in concert with the ER acetyltransferases ATase1/NAT8B and ATase2/NAT8.^7^, ^22^, ^23^, ^24^, ^25^ Their function as lysine acetyltransferases, with their catalytic domain facing the lumen of ER, has been documented in vitro and in vivo, but there is still no evidence of their N‐terminal acetyltransferase activity.^26^, ^27^

On the other hand, van Schaftingen et al. pointed out that NAT8 is exclusively a liver‐ and kidney‐specific enzyme which acetylates cysteine conjugates, but also, less efficiently, some amino acids. It does not need acetyl‐CoA transport in the ER, because its catalytic site is on the cytosolic face of the ER.^28^ The related enzyme, NAT8L, which synthesizes N‐acetylaspartate in the brain, is also an ER enzyme, homologous to NAT8 and it has also its catalytic site facing the cytosol.^28^, ^29^ The enzymes that acetylate the N‐terminus of proteins (NAA10, NAA20, NAA30, NAA80/Nat6) are also cytosolic proteins.^30^, ^31^ Another recent paper also defines NAT8 as liver‐ and kidney‐specific acetyltransferase and demonstrates significant associations between the enzyme encoded by the *NAT8* gene and 14 N‐acetylated amino acids circulating in plasma.^32^ All of this could possibly explain the predominantly normal plasma levels of N‐acetylated amino acids in AT‐1 deficiency. A recently published manuscript describes experiments made in Apicomplexa. Homologs of AT‐1 and NAT8 in *Toxoplasma gondii* and *Plasmodium berghei* were identified and proteome‐wide analyses revealed widespread N‐terminal acetylation marks of secreted proteins in both parasites. However, the authors did not observe a correlation between AT‐1 inactivation and lysine‐ or N‐terminal acetylome variations of ER‐translocated proteins in Apicomplexa which suggested the occurrence of an alternative source of acetyl‐CoA supplying the ER in addition to AT‐1.^33^ Therefore, except for number of papers from Puglielli and his team, there is until now no report on an acetyltransferase in the lumen of the ER that acts on amino acids or on the N‐terminus of a protein.

In conclusion, the therapy regimen was of no benefit. Obviously, AT‐1 has not yet revealed all its secrets and its full function. Considering normal plasma concentrations of N‐acetylated amino acids and CSF concentrations of N‐acetylated amino acids in lower end of the control range, that function could possibly be the brain‐specific N‐acetylation of various amino acids. To better understand the pathomechanism underlying this complex disorder and to design a causal treatment, further studies are needed.

## AUTHOR CONTRIBUTIONS

IB conceived the study and is responsible for the final text of this paper. KŠ contributed to the patient management and wrote major parts of the manuscript. TMAP and UE performed the NGMS analysis and wrote the corresponding sections. DPR, TŽ, SHF, and MD participated in the patient management and organization of testing. TK contributed to neuroradiologic imaging. IB participated in the patient management. KF contributed to laboratory workup. DM, AN, and FRL were involved in gene analysis. TP, UE, and RAW performed and interpreted the biochemical data. All authors reviewed the manuscript.

## FUNDING INFORMATION

This study was partly funded by the financial support for 2020 of the University of Zagreb to Ivo Barić.

## CONFLICT OF INTEREST STATEMENT

The authors declare no conflicts of interest.

## ETHICS STATEMENT

The study has been approved by Ethics Committee of the University Hospital Center Zagreb.

## INFORMED CONSENT

All procedures followed were in accordance with the ethical standards of the responsible committee on human experimentation (institutional and national) and with the Helsinki Declaration of 1975, as revised in 2000. Informed consent was obtained from parents of the patient included in the study.

## ANIMAL RIGHTS

This article does not contain any studies with animal subjects performed by the any of the authors.

## Supporting information


**Table S1:** N‐acetylated amino acids detectable by next‐generation metabolic screening and their presence in cerebrospinal fluid and plasma samples.

## Data Availability

The data that support the findings of this study are available from the corresponding author upon reasonable request.
