# Supplementary material for: Huppke–Brendel syndrome: Novel cases and a therapeutic trial with ketogenic diet and N‐acetylcysteine
Source: JIMD Rep. 2024 Jul 19;65(6):361–70. doi: 10.1002/jmd2.12439 (PMC11540564; doi:10.1002/jmd2.12439)
Supplement: Supplementary file 1 — Table S1: N‐acetylated amino acids detectable by next‐generation metabolic screening and their presence in cerebrospinal fluid and plasma samples. [file JMD2-65-361-s001.docx]

Supplemental table 1. *N-acetylated amino acids detectable by next-generation metabolic screening and their presence in cerebrospinal fluid and plasma samples.*

| **Metabolite** | **CSF** | | | **Plasma** | | |
| --- | --- | --- | --- | --- | --- | --- |
|  | **Adduct** | ***m/z*** | **RT** | **Adduct** | ***m/z*** | **RT** |
| **N6-Acetyllysine** | [M+H]^+^ | 189.1234 | 1.09 | [M+H]^+^ | 189.1234 | 1.06 |
| **N-Acetylalanine** | [M-H]^-^ | 130.0510 | 1.99 | [M-H]^-^ | 130.0510 | 2.1 |
| **N-Acetylasparagine** | [M+Na]^+^ | 197.0533 | 0.92 | nq | - | - |
| **N-Acetylaspartic acid** | [M+Na]^+^ | 198.0373 | 1.22 | nq | - | - |
| **N-Acetylglutamic acid** | [M+H]^+^ | 190.0710 | 1.97 | nq | - | - |
| **N-Acetylglutami­ne** | [M-H]^-^ | 187.0724 | 1.1 | [M-H]^-^ | 187.0724 | 1.2 |
| **N-Acetylglycine** | [M-H]^-^ | 116.0353 | 1.03 | [M+Na]^+^ | 140.0318 | 1.05 |
| **N-Acetylhistidi­ne** | [M+H]^+^ | 198.0873 | 0.84 | nq | - | - |
| **N-Acetylisoleu­cine** | nq | - | - | nq | - | - |
| **N-Acetylleucine** | nq | - | - | nq | - | - |
| **N-Acetylmethio­nine** | [M-H]^-^ | 190.0543 | 4.86 | [M-H]^-^ | 190.0543 | 5.01 |
| **N-Acetylphenyl­alanine** | [M-H]^-^ | 206.0823 | 7.46 | [M-H]^-^ | 206.0823 | 7.65 |
| **N-Acetylproline** | nq | - | - | nq | - | - |
| **N-Acetylserine** | [M+Na]^+^ | 170.0424 | 0.98 | [M+Na]^+^ | 170.0424 | 0.96 |
| **N-Acetylthreonine** | [M+H]^+^ | 162.0761 | 1.74 | [M-H]^-^ | 160.0615 | 1.68 |
| **N-Acetyltyrosine** | [M-H]^-^ | 222.0772 | 5.02 | nq | - | - |
| **N-Acetylvaline** | [M+H]^+^ | 160.0968 | 5.24 | [M-H]^-^ | 158.0823 | 5.13 |
| **Nα-Acetylarginine** | [M+H]^+^ | 217.1295 | 1.11 | [M+H]^+^ | 217.1295 | 1.06 |
| **Nα-Acetylcitrulline** | nq | - | - | nq | - | - |
| **Nα-Acetyllysine** | [M+H]^+^ | 189.1234 | 0.84 | nq | - | - |

*CSF = cerebrospinal fluid, m/z = mass to charge ratio, RT = retention time, nq = not quantifiable, i.e. intensity too low for reliable semi-quantitative analysis for all searched adducts.*
